# Increased Habit Frequency in the Daily Lives of Patients with Acute Anorexia Nervosa

**DOI:** 10.3390/nu14193905

**Published:** 2022-09-21

**Authors:** Maria Seidel, Joseph A. King, Sophia Fürtjes, Natalie Labitzke, Marie-Louis Wronski, Ilka Boehm, Julius Hennig, Katrin Gramatke, Veit Roessner, Stefan Ehrlich

**Affiliations:** 1 Translational Developmental Neuroscience Section, Division of Psychological and Social Medicine and Developmental Neurosciences, Faculty of Medicine, Technische Universität Dresden, 01307 Dresden, Germany; 2 Eating Disorder Treatment and Research Center, Department of Child and Adolescent Psychiatry, Faculty of Medicine, Technische Universität Dresden, 01307 Dresden, Germany; 3 Department of Child and Adolescent Psychiatry and Psychotherapy, Carl Gustav Carus University Hospital Dresden, 01307 Dresden, Germany

**Keywords:** anorexia nervosa, eating disorders, habits, ecological momentary assessment, daily life

## Abstract

Strict eating routines and frequent rigid behavior patterns are commonly observed in patients with anorexia nervosa (AN). A recent theory proposes that while these behaviors may have been reinforced initially, they later become habitual. To date, however, research has been overly focused on eating-disorder (ED)-related habits. Over the course of seven days, we applied an ecological momentary assessment (EMA) to investigate the habit frequency and strength of ED-specific (food intake) and ED-unspecific (hygiene) habits in the daily lives of a sample of *n* = 57 AN and *n* = 57 healthy controls (HC). The results of the hierarchical models revealed that habits were significantly more likely in patients compared with HC for both categories, independently. Furthermore, a lower body mass index (BMI) was associated with increased habit frequency in AN. Our study strengthens the habit theory of AN by showing the relevance of habits beyond ED-specific behavioral domains. This also supports the development of innovative therapeutic interventions targeting habitual behavior in EDs.

## 1. Introduction

“For the past several months, my daughter has been cutting her food into identical little squares, arranging them in a circle on the plate and then eating them slowly in counterclockwise order during every meal”. Strict eating routines and frequent ritualized behavior patterns are commonly observed in individuals suffering from anorexia nervosa (AN). Therapeutic change of these routines and restrictive eating can be challenging [1,2]. Therefore, the importance of improving our understanding of how such behaviors are learned and become persistent and automatic (i.e., habitual) in AN has been emphasized [2,3].

Habits are defined as learned, repetitive, motor, or cognitive behavioral sequences elicited by contextual cues that, once initiated, can be carried out automatically, without conscious oversight [4,5]. Habit formation is thought to follow the principle of stimulus–response learning, according to which (with sufficient repetition) originally goal-directed actions gradually become less sensitive to outcomes [5,6] and, importantly, more closely linked to contextual cues. By shifting from goal-directed to stimulus–response reactions, habitual behavior eventually also becomes less cognitively demanding [7]. Building on this idea, Walsh [2] proposed that AN-related behavior may not be a product of ongoing goal-directed and consciously initiated action, but rather the result of a maladaptive habit formation, i.e., behavior that may have been rewarded initially (e.g., positive social feedback after weight loss), but has become independent from reinforcement over time. The fact that habitual behavior is outcome-independent makes it highly resistant to change, possibly reflecting the treatment resistance often seen in individuals with AN [8]. Therefore, these maladaptive behavior patterns may persist even after weight rehabilitation treatment [9,10,11].

As most research into habits in AN has focused on restrictive eating behavior, it remains unknown whether a tendency toward habitual behavior in AN is specific or domain-independent [12]. The latter would go in line with previous notions of an increased tendency toward rigid routines and strict behavior patterns in response to food restriction [13,14] and in individuals suffering from AN, as well as high rates of compulsive symptoms [15,16,17]. Of note, imbalances between goal-directed and habitual systems have also been implicated in obsessive-compulsive disorder (OCD) [18,19,20], which is also characterized by maladaptive, repetitive, habit-like behaviors and is genetically correlated with AN [21]. Taken together, these previous observations may suggest that individuals with AN generally show more habits in many behavioral domains, unrelated to eating and body weight, possibly due to accelerated habit formation. However, this theory has yet to be confirmed [12,22].

Although the habit theory in AN has enjoyed popularity in recent literature [1,3,12,23,24], only a few studies have tried to investigate more detailed characteristics of these habitual behavior sequences in AN patients [1,25], and experimental evidence is sparse [12]. Supporting the relevance of habits in AN, two studies in adults have found that habit strength (i.e., the degree of behavioral automaticity) of specific food restriction-related behaviors was related to eating-disorder (ED) severity (ED symptom scores), as well as illness duration [1,25]. Interestingly, biological markers that are altered in AN, such as leptin [26], show associations with rigid thinking patterns, such as rumination about food and weight [27]. However, none of these studies have assessed ED-unspecific behaviors, and data were collected only at a single point in time.

Measurement problems have often hindered documenting habitual behavior in real life, as habits have traditionally been assessed as behavioral frequency, which might be difficult to quantify accurately using retrospective methods. Furthermore, studying habits in the laboratory is challenging and several tasks have failed to reliably induce habit-like behaviors [28]. Applying ecological momentary assessment (EMA [29] a method of gathering data from participants in their natural environment in real-time, through a smartphone, using multiple questionnaires a day), might be an advantageous approach to assess and understand habits in AN, because it circumvents many limitations and biases of an artificial laboratory setup, such as inaccurate memory of the exact temporal order of behavioral sequences.

Here, we applied EMA to investigate habit frequency and strength of both ED-specific (food) and ED-unspecific (hygiene) habit categories in a sample of acutely underweight female patients with AN and healthy controls (HC) over the course of seven days. We expected habit frequency and strength for ED-specific and ED-unspecific behavior to be elevated in patients compared with the controls. Furthermore, it was expected that the severity of ED-pathology would be correlated with habit frequency and strength.

## 2. Materials and Methods

### 2.1. Participants

Data were collected from 154 individuals—65 AN patients and a total of 89 HC. For the current analysis, only participants with an EMA compliance rate >40% (i.e., more than 40% of situational data was available) were included, which resulted in the exclusion of 6 AN patients and 16 HC. To account for potential developmental effects and to optimize the comparisons between AN and HC, we implemented a pairwise age-matching algorithm [30] to minimize age differences between individual pairs. The final sample consisted of 57 female patients with acute AN according to Diagnostic and Statistical Manual of Mental Disorders, Fifth Edition (DSM-5) [31] (12.2–24.1 years old) and 57 female HC (12.9–24.3 years old) from Germany. Despite the age-matching procedure, groups showed a small, but significant, age difference (see Table 1) with a mean age difference of 1.6 years between matched pairs. AN patients were admitted to ED programs of a German university child and adolescent psychiatry and psychosomatic medicine department, including a behaviorally oriented nutritional rehabilitation program. Current AN was diagnosed using a modified version of the German expert form of the Structured Interview for Anorexia and Bulimia Nervosa (SIAB-EX [32]) and required a body mass index (BMI) below 17.5 kg/m^2^ (or below the 10th age percentile, if younger than 15.5 years). Information about comorbid diagnoses in AN patients was obtained from the SIAB-EX and medical records, and was confirmed by an expert clinician. To be included in the HC group, participants had to be of normal weight and eumenorrheic. Normal weight was defined as a BMI above 18.5 kg/m^2^ (if older than 16 years)/above the 10th age percentile (if younger than 16 years) and a BMI below 30 kg/m^2^ (if older than 16 years)/below the 97th age percentile (if younger than 16 years). History of any ED or psychiatric disorder was an exclusion criterion. HC were recruited through advertisement among middle school, high school, and university students. Inclusion and exclusion criteria, as well as possible confounding variables, were obtained using semi-structured research interviews—the SIAB-EX [32] and the Mini-International Neuropsychiatric Interview (M.I.N.I. [33]). For further details regarding the diagnostic procedures, see Supplementary Materials File S1.

An a priori power analysis using G*power (Version 3.1., Heinreich Heine Universität Düsseldorf, Germany) [34] on the basis of previously published group differences in disorder-related habit strength between acute AN and HC [25] gauged a sample size of *n* = 17 per group, assuming an effect size that is comparable to the group differences observed in Davis et al. [25] (Cronbach’s alpha = 1.2) and an alpha-error probability of 5% (and a power of 95%).

Study data were collected and managed using secure, web-based electronic data capture tools REDcap (Version 12.0.28, Research Electronic Data Capture, Vanderbilt University, Nashville, TN, USA [35]). This study was approved by the local institutional review board (Institutional Review Board of TU Dresden, Germany, EK536122015), and all participants (if underage, their guardians) gave written informed consent. 

### 2.2. Clinical Measures

To complement the information obtained with the clinical interviews, we assessed ED-specific psychopathology using the Eating Disorders Inventory (EDI-2 [36]), depressive symptoms using the Beck Depression Inventory (BDI-II [37]), trait anxiety symptoms using the State and Trait Anxiety Inventory (STAI/STAI-K (for children) [38]), and obsessive-compulsive traits using a validated German self-report questionnaire to assess obsessive-compulsive traits “Zwangsinventar für Kinder und Jugendliche” (ZWIK [39,40]); for details, see Supplementary Materials File S2.1. The BMI and BMI-standard deviation score (BMI-SDS [41,42]) were measured at baseline one day before starting the EMA assessment. In a subset of the current study sample (*n* = 46 AN and *n* = 56 HC), venous blood for leptin analysis was collected after an overnight fast (see Supplementary Materials File S2.2).

### 2.3. EMA Measures and Materials

Participants were provided with a study smartphone. The app-based questionnaire was designed via an online platform (MovisensXS, Karlsruhe, Germany, library version 4574, www.movisens.com (accessed on 7 August 2022)), which also managed data collection and immediate server upload. When participants received the study smartphone, they were instructed on how to respond to prompts and were briefed on what types of behaviors constituted habits. The EMA asked participants at each prompt whether they had carried out a habit in the last 60 min, and if so, in which of several categories (e.g., food intake, hygiene, or public transport; see Supplementary Materials File S2.1) the activity could be classified. Given that AN patients in the current study were predominantly inpatients, which restricts daily routine, e.g., related to activities in traffic or meal preparation, the focus of the current study was on habits that occurred in one ED-specific (food intake) and in one ED-unspecific (hygiene) category only. Both categories were assumed to be compatible with daily life while participating in a behaviorally-oriented intensive treatment program and were comparable to the data assessed during the daily life of the HC group. After assigning a behavioral sequence to a respective category, participants were asked to write down the habitual behavior in more detail, e.g., for a hygiene habit: “When I brush my teeth I always take the toothbrush with my left hand, apply the tooth paste with my right hand and start brushing my teeth from the left top side, to the left down side, to the front, to the right top side and the right bottom side”. Lastly, participants completed the 10-item Self-Report Habit Index (SRHI [43]) for each reported habit once, so as to assess habit strength, as used in other studies of habitual behaviors in other conditions including EDs [18,25,44]. If the same habit occurred in a previous prompt, the SRHI was not completed again. At each prompt, participants could enter up to five different habits that had occurred since the last prompt. 

### 2.4. Procedure

Participants were initially screened; weighed; interviewed, and, as in our previous studies [45,46], received detailed instructions on how to handle the smartphone, the app, and the content of the questionnaires. For the latter, each participant completed a tutorial, during which they received five example descriptions of habitual behavior sequences of different categories and were asked to fill out the SRHI. They were instructed to answer the questionnaires as soon as the alarm appeared, but were given an additional 30 min after the prompt if they were unable to reply (e.g., during class or work, during mealtimes, or therapy sessions). As in our previous EMA studies [45,46], sampling started the day after screening and lasted for a period of seven days via the signal-contingent assessment method: Alarms occurred at eight semi-random times during a 14 h period that was adapted for each individual to suit different daily routines. Compensation was provided at the end of the study, in accordance with compliance rates.

### 2.5. Statistical Analysis

Because our EMA research design produces nested data [47] in which prompts nest within participants, we conducted two hierarchical generalized linear models (HGLM) via a population-average Bernoulli model for binomial outcome variables (logistic regression for multilevel data) using the software HLM version 8 (Scientific Software International, Inc., Chapel Hill, NC, USA) [48]. These models were chosen as they can account for the correlations within single levels, as well as the interaction between them. The population-average model was favored over the unit-specific model, as we examined the average differences in habit likelihood in each of the two sub-populations [49]. We specified the occurrence of a habit (1 for either food intake (model 1), or hygiene (model 2)) or no habit (0) as a primary outcome measure of interest. The main predictor at the person level was group (1 AN, −1 HC). For both models, age of participant and compliance rate were also entered as control variables, as the groups significantly differed in these variables, which might have influenced reports of habit frequency. Additionally, the variable “trigger”, representing the study progress, was entered at the situation level to account for possible effects of study duration or reactivity to EMA. As habitual behavior might be closely related to compulsions or be different between AN subtypes (restricting or binge-eating/purging), we further validated the main models by (a) excluding two patients with co-morbid OCD and (b) excluding participants with the binge-eating/purging subtype of AN for the models in the supplementary analyses.

Next, to validate the HGLM approach, a variable presenting summarized habit frequency was calculated for each category separately, as the number of times participants answered a prompt and reported a habit that could be assigned to either the food intake (frequency, food) or hygiene (frequency, hygiene) category, divided by the total number of answered prompts. We conducted an independent samples t-test to compare habit frequency between groups. To investigate associations of frequency, food and frequency, hygiene with variables of interest (sensitivity analysis), including BMI-SDS, leptin (i.e., logleptin, see Supplementary Materials File S2.3.), and ED symptoms (EDI-2), as well as with possibly confounding variables such as age, depressive and anxious symptoms (BDI-II; STAI), obsessive-compulsive traits (ZWIK), and duration of illness (DOI), we conducted Pearson correlations and linear regression analysis. Correction for multiple testing was done via the false discovery rate (FDR [50]).

In addition to habit frequency, we also calculated the average habit strength for each category (averaged SRHI, food and SRHI, hygiene) for each participant. As habit strength was not assessed on a momentary level but only once per habit, we used an independent samples t-test to investigate group differences between AN and HC for the average habit strength for each category. 

## 3. Results

### 3.1. Sample

Table 1 summarizes the results of the group comparisons for the demographic and clinical variables. As expected, BMI-SDS and leptin were lower in AN than HC, and patients had higher ED (EDI-2) and depressive (BDI-II), anxious (STAI), and obsessive-compulsive (ZWIK) symptoms. HC were slightly older than AN (see Methods). As in previous studies [46], compliance in EMA was generally good in both groups, but significantly higher in AN than HC, with patients answering on average 83.87% of their prompts compared with 76.35% in HC. For basic sociodemographic characteristics (ethnicity, education, and socioeconomic status), see Supplementary Materials Table S1.

### 3.2. EMA Data

Altogether, participants provided 4729 data points, during which either food intake (*n* = 1458), hygiene (*n* = 1642), or no (*n* = 1629) habit was reported. The results of the HGLMs showed a significant effect of the diagnostic group on habit occurrence for both outcomes (food intake, model 1 and hygiene, model 2). Specifically, the likelihood that a habit was reported was significantly increased in patients with AN compared with HC (food intake: odds ratio (95% confidence interval (CI)): 1.78 (1.35, 2.35)) and hygiene: odds ratio (95% CI): 1.78 (1.16, 1.88); Table 2) when controlling for age, compliance rates, and study progress (i.e., trigger). Excluding two participants with co-morbid OCD diagnosis or 14 AN subtype binge-eating–purging, did not change the results of the main models (Supplementary Materials Tables S2 and S3).

The results of the two hierarchical generalized linear models, as implemented in HLM, used the food habits (model 1) and hygiene habits (model 2) as outcomes. The results reported the coefficients, standard error, and *p* values of the population average model with robust standard errors.

The group differences were confirmed by simple descriptive analyses showing that, over the course of the study, frequency, food and frequency, hygiene were significantly higher in patients with AN compared with HC, whereas the number of prompts that were answered but where no habit was indicated, were not different between groups (Table 1). An exploratory analysis excluding patients that were not treated as inpatients (*n* = 5) had no influence on the statistical significance of the group comparisons. An independent samples t-test did not reveal any general group difference in SRHI, food and SRHI, hygiene (Table 1).

The results of the sensitivity analysis revealed a significant association between frequency, hygiene and BMI-SDS (*r* = −0.357, *p* = 0.006), and a weaker association with leptin in the AN sample (*r* = −0.31, *p* = 0.036), indicating that patients characterized by more severe underweight (and to some degree by lower leptin values, which, however, did not survive correction for multiple testing) displayed a higher number of habits during the week of EMA sampling (Supplementary Materials Table S4). No associations between frequency, food and BMI-SDS (*r* = −0.214, *p* = not significant (n.s.)) or leptin (*r* = −0.062. *p* = n.s.) were observed. Analyses of potentially confounding variables (e.g., age, BDI-II, STAI, ZWIK (including the washing and contamination compulsion subscale), or DOI) did not show any significant correlation with frequency, food or frequency, hygiene, or SRHI, food and SRHI, hygiene (Supplementary Materials Table S4).

## 4. Discussion

The aim of the current study was to investigate ED-specific and ED-unspecific habitual behavior and its potential relationships with ED symptoms and severity in the daily lives of acutely underweight AN patients compared with HC. In line with our hypotheses, patients with AN reported carrying out not only ED-specific (food intake), but also ED-unspecific (hygiene) habitual behavior more frequently than HC. These findings provide evidence that there might be a general tendency for behavior to be guided by habits in AN. 

To the best of our knowledge, this is the first study to apply EMA to a sample of patients with AN to probe habitual behavior in daily life. Using EMA helps circumvent the challenges of assessing these often unconscious, “automatic” behaviors in a laboratory setting (as outlined in the introduction). Supported by two different analytic approaches, our results indicate increased habit frequency, independent of habit category. Moreover, lower BMI-SDS (as well as plasma leptin levels at trend level) in AN patients was associated with an even higher frequency of ED-unspecific habits, indicating a relationship between the severity of the disorder (and degree of undernutrition) and proneness to habitual behaviors. This is in line with previous reports of increased psychological symptoms (depressive, anxious, or OCD symptoms) with lower BMI [51]. Therefore, the current EMA results not only validate previous findings suggesting increased reliance of AN patients on habitual behavior in ED-related behavior patterns (e.g., food restriction, compensatory behavior, and eating rituals [25,52,53]), but also hint toward the importance of habitual behavior in this patient population in general. Moreover, they emphasize the importance of widening the scope to not only focus on a narrow range of behaviors and symptoms of a disorder [54], which has implications for both future research and the development of new therapeutic approaches.

What could the reasons be that some individuals have a stronger tendency to engage in habitual behaviors than others? It has been assumed that habits may have an adaptive function by enabling complex, cognitively costly actions to be carried out with little deliberation, thereby “freeing up” the processing capacity for higher-order strategic tasks [55,56]. For example, there have been suggestions that patients with AN are characterized by increased goal pursuit and proactive control processes [52,57,58,59]. In order to be able to exert such high levels of control successfully, it might be necessary to free up additional cognitive resources by relying on behavior patterns that do not draw upon additional resources. Consequently, the efficiency of habits appears particularly relevant under conditions of heavy load, such as exhaustion or pressure [43]. This also seems to be relevant considering the extreme burden that is associated with the acutely underweight state in patients with AN, which is often associated with neuroendocrinological or metabolic alterations [60], as well as depressive and anxious symptoms [61]. Together, these strains might cause symptomatic patients to operate in their daily lives on a more efficient “habit-driven” mode. If that is the case, we would not expect to see the same pattern in patients following weight restoration or recovery. 

Yet another alternative explanation is based on previous research suggesting that rigid, repetitive eating behavior sequences might help reduce anxiety during mealtimes [62,63]. Indeed, although habits were not related to person-level anxiety symptoms in our sample, such behavioral sequences were found to be elevated in patients who had at least one comorbid diagnosis of anxiety disorder [64]. As rigid, repetitive behavior patterns have been reported to significantly decrease in AN patients after treatment [64,65], it would be interesting to look more closely at the changes in habit frequency in patients with AN during weight gain, as well as momentary associations between anxiety and habitual behavior. Of note, in the current study, habit frequency (of either category) was neither related to obsessive-compulsive symptoms nor did the results change when participants with a comorbid OCD diagnosis were excluded. This finding distinguishes our habit assessment from purely compulsive behavior patterns.

In the present study, we did not find increased habit strength (as assessed with SRHI) in AN patients, neither across nor within the two habit categories. As habitual behavior is acquired through repetition, the frequency of a specific behavior is assumed to be related to the degree of automaticity [66,67]. Previous research by Davis and colleagues has shown that the habit strength of ED-specific habits was stronger in AN patients than HC, and was related to ED symptom severity and illness duration [25]. However, Davis et al. [25] only targeted specific ED-relevant behavior patterns (food restriction, eating delay, compensatory behaviors, and eating rituals) that might not be directly comparable to our “food intake” category and were only assessed once. Additionally, with the item “the respective behavior x is typical me”, the SRHI also extends the definition of habits by connecting it to concepts of the self-identity [68]. It might be that the extent to which eating-related and hygiene habits are part of an individual’s self-identity are different between AN and HC, as the latter has been shown to be altered in AN [69,70].

Two studies in patients with AN using task-based assessments of habits and habit formation have shown heterogeneous results: An outcome-devaluation task designed to measure the conflict of habit learning with goal-directed behavior has not been able to confirm theories of a generally faster habit formation in AN [22]. However, the designs of outcome-devaluation tasks have generally been criticized for tapping predominantly into goal-directed behavior rather than habit formation [28,71]. This might be because training durations in these tasks are often insufficient to instill sufficiently strong habits [28]. Another study used a two-step decision task and a computational model that assessed the degree of model-based (goal-directed) versus model-free (habitual) learning in AN [52]. Rigid behavior patterns across psychiatric disorders have been attributed to an imbalance of the two learning approaches [72,73]. In line with the current results, this imbalance was also found in AN, as model-based learning in a sample of AN patients was decreased compared with HC [52] for both ED-specific (food snack) and ED-unspecific (money) domains. Notions of possible subgroups within AN patients regarding goal-directed vs. habitual aspects of learning might also play a role [74]; however, no such subgroups were visible in the current study. Further research, possibly using other tasks and setups, might be necessary to further establish the role of habits in AN.

Our findings should be interpreted while taking the following limitations into account. First, opportunities to carry out food-related habits might have been different for patients, given the intensive treatment setting. The same limitation might apply to certain (unusual) habits related to hygiene. However, if that was the case, the true group difference would be even larger than the one observed here. Second, because of limitations associated with the inpatient treatment, the exploration of habits of other categories, such as public transportation, was not feasible. Third, our data come from a rather young, non-chronic sample of patients with AN. It might be possible that a longer illness duration (which would translate into more repetitions of specific behavior), or chronicity might increase habit strength [25], which should be taken into account in future research. In addition, the present study did not investigate the possible differences between AN participants with a restrictive vs. binge–purge subtype, but merely confirmed the results in a purely restrictive subsample. On a related note, orthorexia and hygiene-related habits associated with food may constitute a dimension of AN in some individuals; however, this was not formally assessed by our diagnostic procedures. Even though EMA generally entails less memory bias than retrospective measures, one should still be cautious using self-report measures to assess behavior that is assumed to be carried out without consciously initiating it. Lastly, although we identified more habitual behavior in acutely underweight patients with AN, the current study did not allow us to determine whether the found effects were primarily associated with the underweight state (given the correlation with BMI and leptin), or whether they might reflect a trait, possibly playing a role in the etiology and maintenance of the disorder. Therefore, future research should attempt to disambiguate between the state and trait factors. Furthermore, as leptin did not show a strong association with habitual behaviors, it would also be interesting to investigate other nutrition-related parameters such as albumin, zinc, or vitamins [75]. Last, but not least, it might be promising for future research to disentangle the specific association between habitual behavior and ED symptoms more closely, e.g., via different statistical approaches such as network analysis [76], potentially also in other eating disorders such as bulimia nervosa.

## 5. Conclusions

Taken together, using EMA, we found increased habit frequency in ED-specific and ED-unspecific behavior sequences in patients with acute AN. While previous studies in patients with AN using outcome-devaluation tasks designed to measure the conflict of habit learning with goal-directed behavior have not been able to confirm theories of altered habit formation in AN [20], our study strengthens the habit theory in AN by showing the relevance of habits in daily life for these patients beyond the ED symptom level. This also supports the development and application of innovative therapeutic interventions targeting habitual behavior in EDs, for instance habit-reversal training [47], which has been successfully applied to other psychiatric disorders such as OCD [69] or tic disorders [70].

## Figures and Tables

**Table 1:** Descriptive sample characteristics.

Descriptives	*n* (AN/HC)	AN	HC	t	*p*
Age	57/57	15.89 ± 2.05	17.46 ± 3.01	3.25	0.002
BMI	57/57	14.46 ± 1.34	21.01 ± 2.04	20.28	<0.001
BMI-SDS	57/57	−3.33 ± 1.09	−0.03 ± 0.61	19.96	<0.001
Log_10_Leptin	46/56	−0.31 ± 0.80	1.01 ± 0.29	10.60	<0.001
Duration of Illness	57/-	11.66 ± 11.49			
EDI-2	50/57	217.88 ± 43.16	132.65 ± 24.95	−12.69	<0.001
BDI-II	54/57	25.61 ± 10.31	4.98 ± 5.02	−13.51	<0.001
STAI(K)-t	52/57	49.31 ± 12.93	34.16 ± 8.61	7.13	<0.001
ZWIK	51/57	49.25 ± 17.17	38.26 ± 9.93	3.64	<0.001
**Ecological Momentary Assessment**					
Compliance (%)	57/57	83.87 ± 14.15%	76.35 ± 15.57	−2.70	0.008
Frequency, hygiene	57/57	0.40 ± 0.29	0.23 ± 0.15	4.09	<0.001
Frequency, food	57/57	0.39 ± 0.23	0.19 ± 0.14	5.05	<0.001
Prompts accepted but no habit	56/57	13.12 ± 11.95	15.45 ± 11.27	1.07	n.s.
SRHI, hygiene	57/54	66.39 ± 8.34	68.26 ± 8.18	1.19	n.s.
SRHI, food	56/51	56.71 ± 13.08	59.52 ± 10.94	1.19	n.s.

The results of the group comparisons of descriptive data using independent samples t-tests reported the mean, standard deviation, t values, and statistical significance. Frequency, food/hygiene is the total amount of reported food/hygiene habits divided by the total amount of answered prompts over the duration of seven days. *n* = 43 (75.4%) of AN patients were of the restricting subtype. Abbreviations used are as follows: AN = acute anorexia nervosa, BMI = body mass index, BMI-SDS = body mass index standard deviation score, BDI-II = Beck Depression Inventory II, compliance = percentage of answered prompts with a possible total of 56, duration of illness is given in months, EDI-2 = Eating Disorder Inventory 2, HC = healthy control, n.s. = not significant, SRHI = Self-Report Habit Index, STAI (K)-t = State and trait Anxiety Inventory (children version)-trait anxiety score, ZWIK = Zwangsinventar für Kinder und Jugendliche.

**Table 2:** Multilevel estimates for models predicting habit occurrence.

	Model 1: Frequency-Food (*n* = 3087)			Model 2: Frequency-Hygiene (*n* = 3271)		
Parameter	Coefficient	SE	*p*	Coefficient	SE	*p*
Intercept	0.34	0.12	0.006	0.47	0.11	<0.001
Group	0.57	0.13	<0.001	0.39	0.13	0.002
Age	−0.01	0.04	n.s.	−0.02	0.04	n.s.
Compliance	−2.42	0.69	<0.001	−1.49	0.76	n.s.
Trigger	−0.01	0.00	<0.001	−0.01	0.00	<0.001
Trigger × Group	0.00	0.00	n.s.	0.00	0.00	n.s.

## Data Availability

Data are available upon reasonable request from the corresponding author.

## Supplementary Materials

The following supporting information can be downloaded at: www.mdpi.com/article/10.3390/nu14193905/s1. File S1: Participants; File S2: Methods; File S2.1: Clinical and ecological momentary assessment (EMA) measures [39,40]; File S2.2: Blood samples [26]; File S2.3: Statistical Analyses [77,78]; Table S1: Sociodemographic sample characteristics [79]; Table S2: Multilevel estimates for models predicting habit occurrence excluding AN with comorbid obsessive-compulsive disorder (*n* = 2); Table S3: Multilevel estimates for models predicting habit occurrence excluding AN with binge-eating-purging subtype (*n* = 14); Table S4: Associations between habit frequency and clinical variables.
