# Supplementary material for: Increased Habit Frequency in the Daily Lives of Patients with Acute Anorexia Nervosa
Source: Nutrients. 2022 Sep 21;14(19):3905. doi: 10.3390/nu14193905 (PMC9573582; doi:10.3390/nu14193905)
Supplement: Supplementary file 1 [file nutrients-14-03905-s001.zip › nutrients-1881356-supplementary.pdf]

File S1. Participants

Clinical data were also collected from all participants using our own semi-structured research interview which includes several specific questions to assess menstruation history, weight history, general medical and medication history, family psychiatric history, ethnicity, smoking status, and socioeconomic factors (e.g., educational level, occupation, family status, current living situation). Participants of both the acute anorexia nervosa (AN) and healthy control (HC) groups at the focus of the current analyses were excluded if they had a history of organic brain syndrome, schizophrenia, substance dependence, psychosis not otherwise specified (NOS), bipolar disorder, bulimia nervosa or binge-eating disorder. Further exclusion criteria were: IQ <85, psychotropic medication within 6 weeks prior to the study, current substance abuse, inflammatory, neurologic or metabolic illness, chronic medical or neurological illness that could affect appetite, eating behavior, or body weight, clinically relevant anemia, pregnancy or breast feeding.

File S2. Methods

File S2.1. Clinical and ecological momentary assessment (EMA) measures.

During each prompt, participants were asked whether, in the last 60 min, they had carried out a habit, and if yes in which of eight categories the activity could be categorized. Categories were "food preparation", "eating", "hygiene", "styling", "control", "transport", "sport", or "miscellaneous". Due to potential data loss (12–58% reduced sample), "styling", "control" and "miscellaneous" were not included in the analyses.

To assess obsessive-compulsive traits we used the German self-report questionnaire "Zwangsinventar für Kinder und Jugendliche" (ZWIK [39]). The ZWIK is a multidimensional self-report questionnaire and serves the assessment of obsessive-compulsive symptoms in children and adolescents. The 36 items of the questionnaire are grouped into four subscales (contamination thoughts and washing compulsions; control and repetition compulsions; compulsive thoughts regarding harming or injuring others or oneself; counting and questioning compulsions) as well as a total (summary) score. For the ZWIK, high internal consistency for the total score (Cronbach's alpha = 0.92) and sufficient to good internal consistencies for the subscales (Cronbach's alpha = 0.77–0.86) were reported [40].

File S2.2. Blood samples.

In a subset of the current study sample (n = 46 AN and n = 56 HC), venous blood for leptin analysis was collected into vacutainer tubes between 7 and 9 a.m. following an overnight fast within 96h of starting the rehabilitation program. The blood samples obtained were immediately processed as follows: addition of the serine protease inhibitor aprotinin, centrifugation (at ϑ = 5 °C and a = 2,500 ×g for 15 min), aliquotation into Eppendorf Tubes, storage at ϑ = −80 °C until laboratory analysis. Plasma leptin concentrations were measured using a commercially available enzyme-linked immunosorbent assay (ELISA; BioVendor Research and Diagnostic Products, Brno/Czech Republic) with intra- and inter-assay variation coefficients of <10%. Leptin served as an indicator variable for neuroendocrine alterations and nutritional status in AN [26].

File S2.3. Statistical Analyses.

To account for compliance rates in the frequency parameter for each habit category separately, two ratio scores were calculated (Ratio-food, Ratio-hygiene) by dividing the total amount of habits over each category reported by the total amount of answered prompts (independent of whether a habit had occurred or not). For each participant we also calculated average habit strength for both categories (SRHI-eating, SRHI-hygiene) separately.

Leptin concentrations below the lower limit of detection of the applied assay (LOD = 0.20 ng/mL) within the AN sample (n = 12 (26%)) were imputed using a quantile regression multiple imputation approach for left-censored missing data (QRILC). QRILC performs random draws from a truncated distribution with parameters estimated using

quantile regression (derived from the distribution of existing leptin concentrations within detection range; please note that leptin values were log10-transformed and that no further covariates were introduced in the imputation model). QRILC was conducted in R with the help of package "imputeLCMD" [77]. A Gibbs sampler based approach [78] with n = 100 iterative draws per value from the specified truncated distribution was then used to update the initialized values from QRILC and to ensure that the imputed leptin values were positive (on the original scale, i.e., >0) and below LOD.

Table S1. Sociodemographic sample characteristics.

		AN<br>*n* (%)	HC<br>*n* (%)
Ethnicity	European (white)	56 (98.25%)	55 (96.49%)
	Asian	1 (1.75%)	2 (3.5%)
Education	Attending University	2 (3.5%)	14 (24.56%)
	Attending School	53 (92.98%)	42 (73.68%)
	• Gymnasium	40 (75.5%)	39 (92.9%)
	• Realschule	12 (22.6%)	3 (7.1%)
	• Mittelschule	1 (1.9%)	-
	Attending professional training	2 (3.5%)	1 (1.75%)
Living-situation	Living alone	2 (3.5%)	14 (24.56)
	Living with parent(s)	55 (96.49%)	43 (75.44%)
SES (ISCO)	SES	3.55 ± 0.98	3.48 ± 0.99

Table displaying sociodemographic information. AN=acute anorexia nervosa, HC = healthy control, AN = acute anorexia nervosa, SES = socioeconomic status (mean ± standard deviation), which was calculated based on ISCO classification [79] as the participants' SES if living alone, or the highest SES in the family if participant was still living at home. Gymnasium/Realschule/Mittelschule = represent different kind of schools in the German education system with Gymnasium leading to the highest certificate, and Mittelschule the lowest.

Table S2. Multilevel estimates for models predicting habit occurrence excluding AN with comorbid obsessive-compulsive disorder (*n* = 2).

	Model 1:<br>Food			Model 2:<br>Hygiene		
**Parameter**	**Coefficient**	**SE**	***p***	**Coefficient**	**SE**	***p***
Intercept	2.53	0.81	0.002	2.06	0.92	0.027
Group	0.58	0.13	<0.001	0.38	1.46	0.003
Age	−0.01	0.04	n.s.	−0.02	0.04	n.s.
Compliance	−2.54	0.69	<0.001	−1.57	0.76	0.042
Trigger	−0.01	0.00	<0.001	−0.01	0.00	<0.001
Trigger × Group	0.00	0.00	n.s.	0.00	0.00	n.s.

Results of multilevel logistic regression as implemented in HLM, reporting intercept, standard error and p values of the population average model with robust standard errors. The model is identical to the main model (Table 2 in the main text) but excluding *n* = 2 AN participants diagnosed with comorbid obsessive-compulsive disorder. AN = acute anorexia nervosa, HC = healthy control. n.s. = not significant., SE = standard error.

Table S3. Multilevel estimates for models predicting habit occurrence excluding AN with binge-eating-purging subtype (n = 14).

	Model 1: Food			Model 2: Hygiene		
**Parameter**	**Coefficient**	**SE**	***p***	**Coefficient**	**SE**	***p***
Intercept	1.10	0.19	<0.001	1.66	0.97	0.091
Group	0.13	0.03	<0.001	0.38	0.14	0.008
Age	−0.00	0.00	n.s.	−0.01	0.04	n.s.
Compliance	−0.56	0.17	0.002	−1.37	0.86	n.s.
Trigger	−0.00	0.00	<0.001	−0.01	0.00	<0.001
Trigger × Group	0.00	0.00	n.s.	0.00	0.00	n.s.

Results of multilevel logistic regression as implemented in HLM, reporting intercept, standard error and p values of the population average model with robust standard errors. The model is identical to the main model (Table 2 in the main text) but excluding n = 14 AN participants diagnosed with AN subtype binge-easting/purging. AN = acute anorexia nervosa, HC = healthy control. n.s.=not significant.

Table S4. Associations between habit frequency and clinical variables.

	**Frequency-food**	**Frequency-hygiene**
BMI-SDS [a]	−0.21	−0.37 *
Log_{10}Leptin [a]	−0.06	−0.31
EDI-2 [a]	0.13	0.07
BDI-II [b]	0.05	0.09
STAI-trait	−0.09	0.12
ZWIK-total [b]	−0.03	−0.01
ZWIK-Washing [b]	−0.23	−0.06
Age [b]	−0.11	0.01
Duration of illness	−0.06	0.06

Pearson correlation between Frequency-food/Frequency-hygiene (sum of reported habits divided by all answered prompts) and clinical variables for both categories separately in AN patients only; Asterisks denote FDR-corrected significant associations [50], * $p < 0.01$; a = sensitivity, b = confounder analyses and c = exploratory analysis upon reviewer's request; Abbreviations: BMI-SDS = body-mass index standard deviation score, duration of illness = months, Log_{10}Leptin = Log10 of leptin in ng/mL, for values below the limit of detection see section 1.2.3. EDI-2 = Eating Disorder Inventory 2, BDI-II = Beck Depression Inventory, STAI(K) = State and Trait Anxiety Inventory (children's version), ZWIK = Obsessive-compulsive inventory for children, ZWIK-washing = subscale of ZWIK assessing contamination and washing compulsions. To determine whether leptin also explained variance of Frequency-hygiene beyond BMI-SDS, we performed a linear regression with BMI-SDS and orthogonalized Log_{10}Leptin as predictors. Leptin did not explain any additional variance beyond BMI-SDS (beta leptin = −0.15; $p > 0.05$).
